# Hebammen als Influencer bei der Hautpflege atopisch prädisponierter Neugeborener: Eine Umfrage zu den aktuellen Empfehlungen von Hebammen

**DOI:** 10.1007/s00105-024-05334-7

**Published:** 2024-04-19

**Authors:** Petra Staubach-Renz, Sara Schulz, Veronika Weyer-Elberich, Adriane Peveling-Oberhag, Sebastian Zimmer, Joanna Wegner, Anna Sohn, Caroline Mann, Berenice M. Lang

**Affiliations:** 1 Hautklinik Universitätsmedizin Mainz, Langenbeckstr. 1, 55131 Mainz, Deutschland; 2 Institut für Biostatistik und klinische Forschung, Universität Münster, Münster, Deutschland

**Keywords:** Atopische Dermatitis, Primärprävention, Basistherapie, Schulungsprogramme, Hautbarriere, Atopic dermatitis, Primary prevention, Basic skin care, Educational programs, Skin barrier

## Abstract

**Einleitung:**

Die Prävalenz atopischer Erkrankungen ist weiterhin hoch. Erste Studien deuten darauf hin, dass die Primärprävention mit regelmäßiger Basispflege das Auftreten von atopischer Dermatitis bei Säuglingen beeinflussen könnte, allerdings ist die Datenlage nicht ganz eindeutig. Hebammen spielen eine wichtige Rolle bei der Betreuung von Frauen in der Peripartalzeit und damit auch in der Beratung zu Themen wie Hautpflege, Stillen und Ernährung des Neugeborenen und jungen Säuglings. Ziel dieser Studie war es, die Pflegeempfehlungen für Neugeborene durch Hebammen zu ermitteln.

**Methoden:**

Wir führten eine Querschnittsbefragung unter deutschen Hebammen mittels Fragebogen zum Thema Hautpflege von Neugeborenen durch.

**Ergebnisse:**

Es wurden 128 Fragebögen ausgewertet. Die häufigsten Empfehlungen waren pflanzliche Externa auf öliger Basis (34,9 %) und klares Wasser (34,0 %). Etwa 70 % der Hebammen gaben an, verschiedene Optionen zu empfehlen, wenn in der Familie eine atopische Diathese bekannt ist. Bemerkenswert war, dass die meisten Empfehlungen der Hebammen unabhängig vom Vorliegen einer atopischen Diathese dennoch identisch waren. Substanzielle Pflegeprodukte werden nur „bei Bedarf“ verwendet.

**Schlussfolgerung:**

Schulungsprogramme für Hebammen zum Thema „Pflege und Stärkung der Hautbarriere“ unter Berücksichtigung der geltenden Leitlinien zur Allergieprävention sollten durchgeführt werden.

## Abstract (en)

**Introduction:**

The prevalence of atopic diseases remains high. Initial studies suggest that primary prevention with regular basic care may influence the incidence of atopic dermatitis in infants; however, data are unclear. Midwives play an important role in the care of women in the peripartum period and therefore also in providing advice on topics such as skin care, breastfeeding and nutrition of the newborn and young infant. The aim of this study was to determine the care recommendations for newborns by midwives.

**Methods:**

We conducted a cross-sectional survey among German midwives using questionnaires on the topic of newborn skin care.

**Results:**

A total of 128 questionnaires were analyzed. The most common recommendations were oil-based herbal topicals (34.9%) and plain water (34.0%). Approximately 70% of midwives reported recommending various options when there was a known family history of atopic diathesis. It was remarkable that most of the midwives’ recommendations were identical regardless of the presence of an atopic diathesis. Essential care products are only used “when necessary”.

**Conclusion:**

Training programs for midwives on the topic of “care and strengthening of the skin barrier”, taking into account the current guidelines for allergy prevention, should be implemented.

In den westlichen Industrienationen ist weiterhin eine hohe Prävalenz atopischer Erkrankungen (wie allergisches Asthma, allergische Rhinokonjunktivitis oder atopische Dermatitis) zu beobachten, einzelne Manifestationen, wie z. B. die Nahrungsmittelallergien, nehmen seit etwa 10 Jahren weiterhin zu [1]. Die Ursachen hierfür sind bisher nicht vollständig geklärt. Da kausale Therapieansätze begrenzt sind, ist die Primärprävention von besonderer Bedeutung [2–4].

Einige Studien zeigen einen signifikanten Rückgang der atopischen Dermatitis, wenn eine Basistherapie bei Säuglingen von Eltern mit atopischen Erkrankungen zur Primärprävention in den ersten 6 Lebensmonaten angewendet wird [5, 6]. Internationale Experten und Leitlinien empfehlen den Einsatz einer Basistherapie zur Sekundärprävention und Therapie bei allen Patienten, die an atopischer Dermatitis leiden [7, 8].

In der ersten Lebensphase eines Kindes sind in Deutschland Hebammen, Kinderärzte/Kinderärztinnen und Gynäkologen/Gynäkologinnen die wichtigsten Berater für Hautpflegeempfehlungen. Folglich könnten ihre Empfehlungen möglicherweise Krankheitsentstehungen und -verläufe beeinflussen, wie z. B. den Beginn und die Krankheitsaktivität der atopischen Dermatitis.

Derzeit gibt es keine Studien, Richtlinien oder Informationen über die Kriterien, die Hebammen für ihre Hautpflegeempfehlungen verwenden. Ziel dieser Studie war es daher herauszufinden, welche Hautpflegeempfehlungen von Hebammen an Eltern von Neugeborenen in Deutschland gegeben werden.

## Methoden

Wir führten eine Querschnittsstudie durch, um Hebammen in Deutschland zu Hautpflegeempfehlungen zu befragen. Zunächst entwickelten wir einen Fragebogen, an dem 13 interdisziplinäre Experten ([Kinder-]Dermatologen/Dermatologinnen, Gynäkologen/Gynäkologinnen, Kinderärzte/Kinderärztinnen, Hebammen und Apotheker/Apothekerinnen) beteiligt waren. Der Fragebogen besteht aus 8 Fragen zur Hautpflege bei Neugeborenen. Die Antwortmöglichkeiten sind eine Mischung aus Single- und Multiple-Choice-Fragen, die ggf. auch Mehrfachnennungen zulassen, sowie Freitextfeldern.

Die Regionalgruppen des Deutschen Hebammenverbandes wurden gebeten, den Fragebogen per E‑Mail an Hebammen zu verteilen. Es erfolgte keine Rückverfolgung, wie viele Hebammen tatsächlich in Kenntnis des Fragebogens gesetzt wurden. Die Rücksendung aller Fragebögen erfolgte anonym, freiwillig und ohne Aufwandsentschädigung.

Die Studie wurde von der Ethikkommission Rheinland-Pfalz, Deutschland, genehmigt.

Die Auswertung erfolgte mittels deskriptiver Statistik mithilfe von IBM SPSS Statistics (Version 23). Für kategoriale Variablen wurden relative und absolute Häufigkeiten berechnet.

## Ergebnisse

### Studienpopulation

Insgesamt wurden 128 ausgefüllte Fragebögen über einen Zeitraum von 12 Monaten zurückgeschickt. Eine prozentuale Rücksendequote kann aufgrund des Studiendesigns nicht angegeben werden. Die teilnehmenden Hebammen gaben am häufigsten eine Berufstätigkeit von 10 bis 14 Jahren (19,2 %) an, gefolgt von 0 bis 4 Jahren (17,6 %) und 25 bis 29 Jahren (16,0 %), sodass das gesamte Spektrum von Berufsanfängern bis zu erfahrenen Fachkräften vertreten war (Tab. 1). Was den Arbeitsplatz betrifft, so war die Mehrheit der Studienteilnehmerinnen selbstständig (68,0 %), 55,5 % waren (auch) in einer geburtshilflichen Abteilung eines Krankenhauses und 31,3 % in einer Hebammenpraxis beschäftigt (Tab. 2). Es gaben 63,7 % der Hebammen an, dass das Thema Hautpflege Teil ihrer 3‑jährigen Ausbildung war; 66,9 % der Befragten bildeten sich zu diesem Thema im Rahmen von Fortbildungen fort, und 47,6 % der Hebammen bildeten sich durch Lesen von Fachliteratur hierzu fort. Für 88,7 % war das „Sammeln von Erfahrungen“ und für 64,0 % der „kollegiale Austausch“ von Relevanz.

**Tab. 1:** Informationen über die Jahre der Berufserfahrung der Umfrageteilnehmer (*n* = 125)

Jahre der Berufserfahrung	*n* (%)
0–4	22 (17,6)
5–9	17 (13,6)
10–14	24 (19,2)
15–19	11 (8,8)
20–24	12 (9,6)
25–29	20 (16,0)
30–34	10 (8,0)
35–39	5 (4,0)
40–44	3 (2,4)
45–49	1 (0,8)

**Tab. 2:** Angaben zum Arbeitsplatz der Umfrageteilnehmer (*n* = 128), Mehrfachnennungen möglich

Arbeitsplatz	*n* (%)
Selbstständig	87 (68,0)
Abteilung „Geburtshilfe“ im Krankenhaus	71 (55,5)
Hebammenpraxis	40 (31,3)
Hebammen-geführter Kreißsaal	6 (4,7)
Geburtshaus	2 (1,6)

### Empfohlenes Verhalten und Bewusstsein für atopische Krankheiten bei Eltern

Zunächst wurden die Hebammen gefragt, ob sie den Eltern von Neugeborenen generell Ratschläge zur Hautpflege der Kinder geben. Es gaben 70,3 % an, dass sie dies generell tun, 28,1 % nur auf Nachfrage, und nur 1,6 % antworteten, dass sie überhaupt keine Empfehlungen zu diesem Thema geben. Anschließend wurden die Hebammen gefragt, welche Aspekte sie bei ihren Hautpflegeempfehlungen berücksichtigen und ob die medizinische Vorgeschichte der Eltern wie atopische Diathese eine Rolle in der Empfehlung für die Kinder spielt. Es gaben 70,4 % der Hebammen an, bekannte Erkrankungen der Eltern in die Hautpflegeempfehlungen der Kinder einzubeziehen (Gruppe 1); 29,6 % gaben an, die Erkrankungen der Eltern bei der Beratung nicht zu berücksichtigen (Gruppe 2). Anschließend analysierten wir die Antworten der Hebammen aus den beiden Gruppen, um festzustellen, ob spezielle Aspekte der Eltern, wie beispielsweise atopische Erkrankungen, einen Einfluss auf die Hautpflegeempfehlungen der Kinder haben.

### Startpunkt der Hautpflege und Häufigkeit der Anwendung

**Abb. 1:**
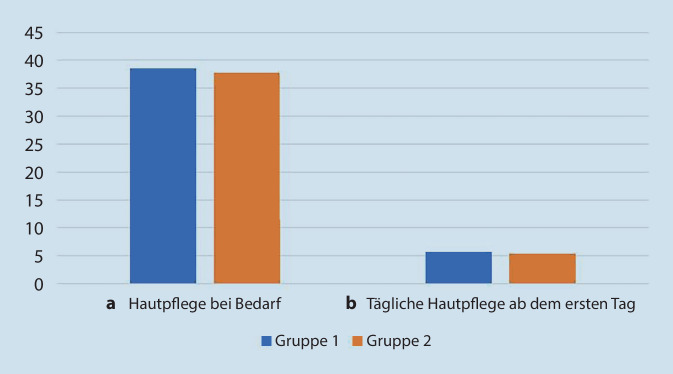
**a** Startpunkt der Hautpflegemittel im Vergleich zwischen Gruppe 1 und Gruppe 2. **b** Häufigkeit der Anwendung von Hautpflegemittel im Vergleich zwischen Gruppe 1 und Gruppe 2

Die Hebammen wurden gefragt, zu welchem Zeitpunkt nach der Geburt sie empfehlen, mit der Hautpflege zu beginnen (Tab. 3). Die Mehrheit in beiden Gruppen gab an, dass sie Hautpflege bei Bedarf empfiehlt, d. h. nur, wenn die Neugeborenen Hautläsionen aufweisen (38,6 % in Gruppe 1, 37,8 % in Gruppe 2) (Abb. 1a). Allerdings war in Gruppe 1 der Prozentsatz der Hebammen, die die Verwendung von Pflegeprodukten innerhalb der ersten Lebenswoche empfahlen, höher als in Gruppe 2 (14,8 % vs. 5,4 %).

**Tab. 3:** Empfehlungen für den Beginn der Hautpflege, aufgeteilt in Antworten der Gruppe 1 („Erkrankung der Eltern wird berücksichtigt“) und Gruppe 2 („Erkrankung der Eltern spielt keine Rolle“) (Anzahl und Prozentsatz der Hebammen)

	Gruppe 1; *n* = 88 *n* (%)	Gruppe 2; *n* = 37 *n* (%)
Hautpflegeprodukte bei Bedarf	34 (38,6)	14 (37,8)
Waschen mit Wasser bei Bedarf	6 (6,8)	6 (16,2)
Verwendung von Pflegeprodukten sofort bis zum 7. Tag nach der Geburt	13 (14,8)	2 (5,4)
Waschen mit Wasser und Anwendung von Pflegeprodukten sofort bis zum 7. Tag nach der Geburt	7 (8,0)	2 (5,4)
Waschen mit Wasser sofort bis zum 7. Tag nach der Geburt	7 (8,0)	6 (16,2)

Dann wurden die Hebammen gefragt, wie oft pro Woche sie die Hautpflege empfehlen. Die Mehrheit in beiden Gruppen gab an, dass sie empfehlen die Pflegeprodukte wöchentlich nach Bedarf und nicht nach einem festen Zeitplan anzuwenden (Tab. 4; Abb. 1b). Es wurden keine Unterschiede zwischen den Gruppen festgestellt, was zeigt, dass unabhängig von der Familienanamnese dieselben Empfehlungen gegeben wurden (30,7 % in Gruppe 1 und 29,7 % in Gruppe 2). Der Anteil der Empfehlungen, sich täglich nur mit klarem Wasser zu waschen, war in Gruppe 2 höher (6,8 % in Gruppe 1 vs. 16,2 % in Gruppe 2).

**Tab. 4:** Häufigkeit der Empfehlung für tägliche und wöchentliche Hautpflege Gruppe 1/Gruppe 2 (Anzahl und Prozentsatz der Hebammen)

	Gruppe 1; *n* = 88 *n* (%)	Gruppe 2; *n* = 37 *n* (%)
Täglich undefiniert	8 (9,1)	2 (5,4)
Tägliche Anwendung von Hautpflegeprodukten	2 (2,3)	1 (2,7)
Tägliches Waschen mit Wasser	6 (6,8)	6 (16,2)
Tägliches Waschen mit Wasser und Anwendung von Hautpflegeprodukten	3 (3,4)	1 (2,7)
Wöchentlich nach Bedarf Anwendung von Hautpflegeprodukten	27 (30,7)	11 (29,7)
1- bis 3‑mal wöchentlich Anwendung von Hautpflegeprodukten	8 (9,1)	0 (0,0)
1- bis 3‑mal wöchentlich Anwendung von Hautpflegeprodukten nach dem Baden	10 (11,4)	3 (8,1)
Wöchentlich Waschen mit Wasser nach Bedarf	4 (4,5)	6 (16,2)

### Empfohlene Art der Hautpflege

Zudem wurden die Hebammen gefragt, ob sie von sich aus Hautpflegeprodukte für Neugeborene nach der Geburt empfehlen und, wenn ja, welche Art von Hautpflege sie empfehlen. Die Mehrheit der Hebammen empfiehlt pflanzliche Externa als erste Wahl (44,4 % in Gruppe 1 und 45,9 % in Gruppe 2). Die bevorzugte Textur war eine ölige Grundlage (23,9 % in Gruppe 1 und 40,5 % in Gruppe 2), obwohl die Hebammen in Gruppe 1 mehr Wert auf die Textur der Grundlage zu legen scheinen („pflanzliche Externa unterschiedlicher Textur“ 20,5 % in Gruppe 1 vs. 5,4 % in Gruppe 2). Auch hier neigten die Hebammen der Gruppe 2 dazu, nur klares Wasser zu verwenden (40,5 % in Gruppe 2). Muttermilch war in beiden Gruppen üblich (Tab. 5). Auf die Frage, ob Hebammen bei den von ihnen empfohlenen Hautpflegeprodukten auf besondere Faktoren achten, hielten 36,9 % der Studienteilnehmerinnen den Fett- und Wassergehalt für wichtig.

**Tab. 5:** Beratung über Hautpflegeprodukte für Neugeborene nach der Geburt Gruppe 1/Gruppe 2 (Anzahl und Prozentsatz der Hebammen)

	Gruppe 1; *n* = 88 *n* (%)	Gruppe 2; *n* = 37 *n* (%)
Pflanzliche Externa auf öliger Basis	21 (23,9)	15 (40,5)
Pflanzliche Externa unterschiedlicher Textur	18 (20,5)	2 (5,4)
„Weniger ist mehr“/gar nichts tun	8 (9,1)	1 (2,7)
Klares Wasser	19 (21,6)	15 (40,5)
Muttermilch zur Hautpflege	13 (14,8)	4 (10,8)

## Diskussion

Die dermatologische Basistherapie bei atopischen Menschen gewinnt zunehmend an Bedeutung. Aktuelle Forschungen zum Thema Basistherapie haben bisher unterschiedliche Effekte gezeigt [6, 9, 10], sodass einheitliche Empfehlungen hinsichtlich der Primärprävention der atopischen Dermatitis noch nicht in der Leitlinie Allergieprävention [8] enthalten sind. Da aber frühes Eincremen mit geeigneten Externa, die möglichst kein Protein von potenziell allergenen Nahrungsmitteln oder Kontaktantigenen enthalten sollten, die Hautbarriere stärken könnte – gerade bei Neugeborenen atopischer Eltern –, ist zu überlegen, ob die Empfehlung, in den ersten 6 Lebensmonaten täglich Basistherapeutika anzuwenden, nicht sinnvoll sein könnte und zumindest nicht schädlich wäre [5, 6]. Weitere Studien hierzu werden weltweit durchgeführt [9, 11], um den Stellenwert und schlussendlich die Empfehlung für oder gegen eine Primärprävention durch Externa für Betroffene und Eltern zu vereinheitlichen.

In unserer Kohorte gaben 70,4 % der Hebammen an, dass das Vorhandensein von atopischen Erkrankungen bei den Eltern einen Einfluss auf die Hautpflegeempfehlungen hat. Im Gegensatz dazu berücksichtigt fast ein Drittel der Hebammen die atopische Prädisposition bei den Behandlungsempfehlungen nicht. Ob bei der Risikogruppe atopisch prädisponierter Neugeborener die frühzeitige und regelmäßige Basistherapie wirklich präventiv ist, werden die noch laufenden Studien hoffentlich bald zeigen, sodass es einheitlichere Empfehlungen geben wird. Überraschenderweise konnte gezeigt werden, dass beide Gruppen unabhängig von der atopischen Veranlagung im Sinne der Primärprävention die gleichen Hautpflegemaßnahmen empfehlen. Insgesamt gibt es eine große Anzahl von Hebammen, die Hautpflegeprodukte nur empfehlen, wenn die Babys klinisch relevante Hautläsionen aufweisen.

Unsere Forschungsfragestellung bezog sich auch darauf, welche Arten von Formulierungen und Inhaltsstoffen von Hebammen bevorzugt und daher empfohlen werden. Die meisten Empfehlungen waren pflanzliche Externa in öligen Vehikeln oder reine Pflanzenöle. Außerdem waren klares Wasser oder Muttermilch die bevorzugten Antworten. Rein natürliche Produkte scheinen immer beliebter zu werden, obwohl sie – abhängig von der Qualität der Inhaltsstoffe – nachweislich sensibilisierende Stoffe enthalten, unterschiedlich zusammengesetzt sind und Auslöser einer Kontaktdermatitis sein können [12, 13]. Dieser Aspekt muss bei der Beratung der Eltern berücksichtigt werden, und es sollte zumindest eine Aufklärung über sensibilisierende Inhaltsstoffe oder mögliche Entstehung von Allergien erfolgen. Nationale und internationale Leitlinien empfehlen die Kombination von feuchtigkeitsspendenden Inhaltsstoffen wie Glycerin in Verbindung mit rückfettenden Inhaltsstoffen wie natürliche Öle sowie Ceramide, die die Barrierestruktur und -funktion positiv beeinflussen, als lehrbuchmäßige Basistherapie für eine proaktive und präventive therapeutische Option zur Stärkung der Hautbarriere primär bei Frühzeichen und der Behandlung einer atopischen Dermatitis [7, 8, 14]. Individuelle Unterschiede im klinischen Erscheinungsbild der Erkrankung und der Haut sowie der Wechsel der Jahreszeiten sind zu berücksichtigen. Ebenso muss die Haut regelmäßig beurteilt werden. Fettige Inhaltsstoffe allein sind nicht zu empfehlen [7, 15, 16]. In unserer Studie berücksichtigen nur 36,9 % der Hebammen in ihren Empfehlungen die Kombination von Fett- und Wassergehalt in Pflegeprodukten. Unsere Daten zeigen, dass das Wissen über das gesamte Spektrum an Formulierungen und Inhaltsstoffen für eine optimale Hautpflege aus dermatologischer Sicht in der täglichen Praxis der Hebammen nicht immer vorhanden zu sein scheint.

Unsere Daten zeigen, dass das Bewusstsein für die Risikogruppe der Neugeborenen atopischer Eltern zwar vorhanden ist, aber die Umsetzung in der täglichen Praxis fehlt. Dies begründet sich auch daher, dass es zur Primärprävention der atopischen Dermatitis keine einheitlichen Empfehlungen gibt, da die Datenlage noch kontrovers ist, obwohl die Stärkung der Hautbarriere sicher ein Target zur Primärprävention von atopischer Dermatitis und Nahrungsmittelallergien sein könnte [17, 18]. Um dies zu ändern, ist die Integration neuer Ausbildungsinhalte zum Thema Basispflege in der Hebammenausbildung künftig unerlässlich. Nur etwa 64 % der Hebammen gaben an, dass Hautpflege Teil ihrer Ausbildung ist und dass es an Aktualisierungen mangelt. Obwohl die Fortbildung für Hebammen obligatorisch ist, scheint die Hautpflege nicht Teil des Lehrplans zu sein und wurde nur von etwa 67 % genutzt. Wie lange die letzte Fortbildung zum Thema Hautpflege zurückliegt, wurde nicht abgefragt, sollte aber in künftigen Erhebungen unbedingt mitberücksichtigt werden. Erweiterte Kenntnisse in der Basistherapie sind für Hebammen wichtig; sie sollten in der Lage sein, die Eltern betroffener Kinder richtig zu beraten. Nur gut informierte Eltern sind in der Lage, die Therapie richtig durchzuführen, um eine Verbesserung der Hautbarrierefunktion und damit eventuell eine Verringerung von atopischer Dermatitis und Nahrungsmittelsensibilisierungen zu erzielen [5, 6, 15].

Einer der Schwachpunkte unserer Studie ist die geringe Rücklaufquote der Umfrage unter den Hebammen und damit die Gefahr, dass die beantworteten Fragebögen nicht repräsentativ sind. In Deutschland gibt es etwa 20.000 Hebammen, die Mitglied im Deutschen Hebammenverband sind [19]. Der Fragebogen wurde von den Regionalgruppen per E‑Mail an die Mitglieder verteilt. Nur 128 Fragebögen wurden zurückgeschickt. Ein Grund dafür könnte sein, dass E‑Mail-Kommunikation nicht die beste Benachrichtigungsmethode ist und wir die Fragebögen per Post hätten zustellen sollen. Ein anderer Grund könnte sein, dass es einfach zu wenig Bewusstsein dafür gibt, dass Information, Aufklärung und Forschung in diesem Bereich wichtig sind und dass die von Hebammen gesammelten Daten für die Versorgungsforschung relevant sind. In Anbetracht dessen scheint die künftige Integration dermatologischer Themen ein wesentliches Instrument für die Aus- und Weiterbildung zu sein. Ähnliche Probleme scheinen auch in anderen medizinischen Berufen zu bestehen. Leider ist die Beurteilung des Hautzustandes mit der richtigen Wahl eines geeigneten Hautpflegemittels in vielen Berufsgruppen in Deutschland eine große Herausforderung. Auch Krankenpflegekräfte scheinen mehr Fortbildung zu benötigen, einschließlich Auffrischungen [20]. Neben Hebammen und Krankenpflegekräften sind Kinderärzte/Kinderärztinnen die wichtigsten Gesundheitsdienstleister für Neugeborene, und auch in dieser Berufsgruppe sollten weitere Schulungen durchgeführt werden.
